# Academic Goal Profiles and Learning Strategies in Adolescence

**DOI:** 10.3389/fpsyg.2018.01892

**Published:** 2018-10-08

**Authors:** María Carmen Martínez-Monteagudo, Beatriz Delgado, Ricardo Sanmartín, Candido J. Inglés, José Manuel García-Fernández

**Affiliations:** ^1^Department of Developmental Psychology and Didactic, Faculty of Education, University of Alicante, Alicante, Spain; ^2^Department of Health Psychology and Didactic, Faculty of Psychology, Miguel Hernández University, Elche, Spain

**Keywords:** academic goals, motivational profiles, learning strategies, latent class analysis, adolescence

## Abstract

The objective of this study was to verify whether or not a combination of academic goals may be established in different profiles of high school students. Subsequently, the study examined if statistically significant differences exist between the profiles obtained with respect to learning strategies used by the students. The Achievement Goal Tendencies Questionnaire (AGTQ) and the Learning and Studies Skills Inventory-High School Version (LASSI-HS) were administered to a sample of 2,069 high school students aged 12–16 (*M* = 14.11; *SD* = 1.35) and which was formed by 1,073 girls and 996 boys. Four academic goal profiles were identified using latent class analysis: a group of students with a high academic goal (HAG) profile (668 students), a group of students with a low academic goal (LAG) profile (502 students), a group of students with a predominance of learning goals and achievement goals (LGAG) (489 students) and a final group of students with a predominance of social reinforcement goals and achievement goals (410 students). The results revealed statistically significant differences between the profiles obtained with respect to learning strategies because students from the combined LGAG and HAG profiles used more learning strategies that those in the LAG and Achievement Goals and Social Reinforcement (AGSR)groups. However, the relationship between these motivational profiles and the obtainment of a higher academic performance has not been proven and it should be the subject of study in future research. Consequently, this study can be used to help in the development of strategies and intervention programs to promote the use of multiple academic goals in high school students.

## Introduction

The motivation driving students to learn has been one of the most widely studied variables in the fields of psychology and education. Within the motivational context, the academic goals pursued by students have been considered a key factor to ensure satisfactory academic adjustment (Elliot, 2005). Thus, traditionally, empirical evidence has suggested that certain students seek to acquire academic knowledge and improve their skills (learning goals), while other students only pursue the achievement of positive assessments of their ability and attempt to avoid negative evaluations of themselves (performance goals) (Elliot, 2005). Performance goals have been divided in turn into social reinforcement and achievement goals. Therefore, the motivation to learn from students with social reinforcement goals is primarily to obtain the approval of and avoid the rejection by parents and teachers, whereas students with achievement goals pursue high academic qualifications and progress in their studies. This has been the traditional conception of most of the scientific studies conduced with respect to students’ academic goals, thus, it has been considered that students are exclusively classified into one of the two mentioned categories (learning goals vs. performance goals). However, in the last decade, it has been considered that students may pursue different goals during their learning process, with these goals not necessarily being mutually exclusive (Linnenbrink, 2005; Daniels et al., 2008; Valle et al., 2010; Wormington et al., 2012; Hofer and Fries, 2016; Navas et al., 2016; Wormington and Linnenbrink-García, 2017; Clarence, 2018; Ning, 2018; Rameli et al., 2018). Thus, the same student can pursue different goals depending on the learning activity proposed in the classroom or depending on the specific subject. So, unattractive learning activities aside, reasons distinct from an intrinsic interest in the task may motivate the student (obtaining good academic grades, recognition of parents and teachers or receiving rewards), which may be powerful incentives to promote and maintain academic commitment. Dweck (2001) mentions that the diverse school situations enable students to simultaneously achieve different aims and to pursue multiple goals at the same time.

In fact, more and more studies indicate a higher academic performance by those students who present different combinations of goals in specific school situations (Pintrich, 2000; Wentzel, 2000; Valle et al., 2003b, 2016; Daniels et al., 2008; Berger, 2012). The theory of multiple academic goals also provides a convincing explanation of the existing divergences with respect to the advantages and disadvantages of adopting certain types of goals as opposed to others.

So, Valle et al. (2003a) using the Achievement Goal Tendencies Questionnaire (AGTQ; Hayamizu and Weiner, 1991) on a sample of 609 university students, identified three profiles of different goals. A group of students with a predominance of multiple goals, a group of students with a predominance of achievement goals and a third group of students with a predominance of learning goals. Upon further investigation and also using a college sample, Valle et al. (2010) identified six different motivational goal profiles: (a) low profile widespread motivation; (b) profile oriented toward avoiding presenting a bad image to others; (c) learning-oriented; (d) learning-oriented and avoiding presenting a bad image to others; (e) learning-oriented and achieving better academic results than the others; and (f) general high motivation profile. With a teenaged sample, Valle et al. (2009) identified four groups: (a) learning-oriented and achievement profile; (b) generalized profile with high motivation (high scores on all goals evaluated); (c) prevalence of fear of failure; and (d) generally low motivation (low scores on all goals evaluated). Ning (2018) on a sample of Singapore primary students identified six types of students with distinct patterns of achievement goal motivation: high goal-oriented (strong multiple goals), average goal-oriented (moderate multiple goals), low goal-oriented (weak multiple goals), performance and approach-oriented (high mastery- and performance-approach, high performance-avoidance, low mastery-avoidance), approach-oriented (high mastery- and performance-approach, low mastery- and performance-avoidance), and mastery-oriented (moderate mastery-approach and mastery-avoidance, low performance-approach, and performance-avoidance). Thus previous works consider (Valle et al., 2003a, 2009, 2010; Linnenbrink, 2005; Daniels et al., 2008; Wormington et al., 2012; Hofer and Fries, 2016; Navas et al., 2016; Wormington and Linnenbrink-García, 2017; Clarence, 2018; Ning, 2018; Rameli et al., 2018), providing data that reveal the existence of specific motivational goal profiles, although depending on the assessment tool used, the number and composition of the groups may vary. Moreover, as mentioned above, most of these studies use sample of university students.

The comparison of results with respect to the motivational profiles found in the university samples and the samples of adolescents is complex, because the evolutionary moment of the student can influence their academic goals. Thus, for example, social recognition is especially relevant in the adolescent stage, being able to complicate the control of multiple academic goals. The goals of social assessment, for their part, make the student strive in their studies based on the obtainment of rewards, praises directed to them and the evaluations they receive from others (peers and family), which may displace other goals. However, the characteristics of university education are different, due to the future impact of academic performance, the student’s level of maturity, etc.

As for the method used to classify goal profiles, it is important to mention that until now previous studies have made use of traditional techniques, such as the case of median split or cluster analysis (Seifert, 1995; Turner et al., 1998; Shih, 2005; Wilson et al., 2016). However, a latent variable mixture (latent class analysis, latent profile analysis mixed Rasch model, among others) has been proposed as an appropriate person-centered analysis since it allows for the comparison of models with different number of classes, considering the measurement mistake made during the classification process (Vermunt and Magidson, 2002; Lubke and Muthén, 2005).

On the other hand, empirical evidence has shown that students who pursue goals are closely related to the learning strategies used (Wolters et al., 1996; Elliot et al., 1999; Radosevich et al., 2004; Bråten and Olaussen, 2005; Liem et al., 2008; Lüftenegger et al., 2016; De la Fuente et al., 2017). Actually, deep learning strategies are usually found in individuals who enhance their skills and appreciate both learning and intend to learn (Valle et al., 2003a,b). However, the relationship between goal performance and commitment to learning seems to be more complex (Brophy, 2005). Although these goals are often related to a low use of deep processing strategies (Pintrich and García, 1991; Seifert, 1995), in some studies they have been associated with high academic scores (Elliot, 1999; Harackiewicz et al., 2000; Midgley et al., 2001; Shih, 2005) and the use of learning strategies (Wolters et al., 1996; Wolters, 2004). As Brophy (2005) suggests, more research is needed in order to clarify the role of goal performance in learning and academic achievement. Although theoretically it is assumed that the adoption of such targets leads to low level and superficial learning, on a practical level, researchers often end up recommending professionals in the education classes to promote the two types of goals, since this combination tends to offer the best results. Thus, when students proposed study, even in order to get good grades or to avoid negative judgments about them, the use of learning strategies is favored.

Thus, although research has considered academic goals as being mutually exclusive, learning goals are related to an increase in the use of learning strategies, while performance goals are related to a decline in the use of these same strategies. However, when it is assumed that students can pursue multiple goals depending on school situations, it has been noted that learners with high levels of the different types of goals reveal a more adaptive pattern. Not only do they use more learning strategies leading to deep processing of information (Bouffard et al., 1995; Piñeiro et al., 2001; Valle et al., 2003b; Daniels et al., 2008; Ahmad and Bashir, 2009), but they also show higher levels of motivation, self-concept, self-regulation strategies, academic self-attributions and academic performance (Bouffard et al., 1995).

The aim of this study is to verify the possibility of obtaining different motivational profiles depending on the weight of each of the academic goals pursued by the students. Thus, taking into consideration the results provided by the mentioned works, we expect to find that the combinations of academics goals will lead to different motivational profiles. Secondly, after finding and defining the motivational profiles, we will attempt to determine whether or not significant group differences exist with regard to the learning strategies. Specifically, it is expected that students with a high academic goal (HAG) profile will obtain significantly higher scores on learning strategies than the remaining groups.

## Materials and Methods

### Ethics Statement

All standards for research with human subjects were respected, in accordance with the ethical principles of the Helsinki Declaration and the Ethics Committee of the University of Alicante. The protocol was approved by the Ethics Committee of the University of Alicante. The parent or legal guardians of all participants gave written informed consent in accordance with the Declaration of Helsinki.

### Participants

The cluster sampling method was used to recruit the sample of participants. The primary sampling units were the provinces of Alicante and Murcia and each of the provinces were divided into five different regions (north, south, center, east and west). The secondary units, which were the Compulsory Secondary Education Centers, were randomly chosen from each of the regions and between 1 and 3 centers were selected from each region depending on the population density of the analyzed region. Finally, the tertiary units were the classrooms, which were obtained by random choosing one class per educational level (between 1st and 4th grade of Compulsory Secondary Education) from each of the selected centers. The total number of participants recruited was 2,141 Spanish high school students from 1st to 4th grade of Compulsory Secondary Education, of whom 72 (3.4%) did not participate in the study due to mistakes made during the fulfillment process or failing to present the legal guardians’ consent to collaborate in the investigation. The final sample included 2,069 students (1,073 girls and 996 boys; see **Table 1**), whose age ranged between 12 and 16 years (*M* = 14.11; *SD* = 1.35). The ethnic composition of the sample was: 90.1% Spaniards, 6.81% Latin American, 2.12% European, 0.62% Asian, and 0.35% Arab. Using the χ^2^ test of homogeneity of the frequency distribution, we verified the absence of statistically significant differences among the groups of Sex × Age χ^2^ = 3.69, *p* = 0.361).

**Table 1:** Distribution of the sample by sex and educational level.

Sex	Educational level				Total
	1st Grade of CSE	2nd Grade of CSE	3rd Grade of CSE	4th Grade of CSE	
Boys	317	252	264	219	1052
	15.40%	12.20%	12.80%	10.5%	50.90%
Girls	270	264	249	234	1017
	13.1%	12.8%	12.00%	11.30%	49.10%
Total	587	516	513	453	2069
	28.50%	24.90%	24.80%	21.80%	100.00%

*CSE, Compulsory Secondary Education.*

### Measures

*Achievement Goal Tendencies Questionnaire* (AGTQ; Hayamizu and Weiner, 1991; adaptation of Inglés et al., 2011). The test contains 20 items that are rated on a 5-point Likert scale (1: *never;* 5: *always*) to obtain the analysis of three tendencies or goal orientations (*Learning, Achievement* and *Reinforcement Goals*). Firstly, *Learning Goals* consists of 8 items and refers to the students’ attitude toward learning which is based on enhancing their knowledge and improving their competences. Secondly, *Achievement Goals* consists of 6 items and shows the students’ inclination to learn in order to obtain high marks and achieve academic success. Finally, *Social Reinforcement Goals*, which is made up of 6 items, reveals the students’ attitude toward learning based on obtaining parent and teacher approval. The AGTQ is one of the most widely used questionnaires for the measurement of academic goals, demonstrating its adequate psychometric properties (Inglés et al., 2011). In this study, the various subscales of the questionnaire demonstrated their adequate reliability with Cronbach’s alpha values equaling 0.86 for Learning Goals, 0.75 for Achievement Goals, and 0.89 for Social Reinforcement Goals.

*Learning and Studies Skills Inventory-High School Version* (LASSI-HS; Weinstein and Palmer, 2002). The LASSI-HS is a self-report instrument designed to assess strategies and skills developed by high school students in academic settings. It consists of 76 items and 10 subscales: *Attitude, Motivation, Time Management, Anxiety, Concentration, Information Processing, Selecting Main Ideas, Study Aids, Self-Testing* and *Test Strategies*, by which students assess their own way of studying. Each subscale is related to one of the three components of strategic learning: cognitive ability, willingness and self-regulation (Weinstein and Palmer, 2002). However, this study has evaluated each subscale separately, in accordance with the recommendations of the original authors and not this three-factor model which has been found psychometrically (Cano, 2006). Each subscale contains 5–8 items, with each item responded to on a 5-point scale ranging from 1 (*describes me a lot*) to 5 (*does not describe me at all*). The reliability of the instrument has been confirmed by the original authors (Weinstein and Palmer, 2002) and by Spanish researchers (Núñez et al., 1998). In this study, the internal consistency coefficients (Cronbach’s alpha) were adequate for all of the subscales (0.78 for Attitude, 0.76 for Motivation, 0.72 for Time Management, 0.71 for Anxiety, 0.82 for Concentration, 0.75 for Information Processing Scale, 0.75 for Selecting Main Ideas, 0.72 for Study Aids Scales, 0.77 for Self-Testing Scale, and 0.78 for Test Strategies).

Besides, in **Table 2**, it can be observed in previous studies and in the present study using both questionnaires: the age of the samples in which it have been administered, the average variances extracted, the Cronbach’s Alpha and the Omega values.

**Table 2:** Factorial analyses, average variances extracted, Cronbach’s alpha and omega values for previous studies and the present studies using the Achievement Goal Tendencies Questionnaire and the Learning and Studies Skills Inventory-High School Version.

Authors	Age	Factorial analyses	Average variances extracted	Cronbach’s alpha	Omega
**AGTQ**					
Hayamizu and Weiner, 1991	+18	EFA	Total = 52.4%, LG = 23%, SRG = 14.9% and PG = 14.8%	0.89 (LG), 0.78 (SRG), and 0.71 (PG)	–
Núñez and González-Pienda, 1994	10–14	–	–	0.93 (Total AGTQ), 0.85 (LG), 0.84 (SRG), and 0.88 (PG)	–
García et al., 1998	10–14	EFA	Total = 56.8%. LG = 34.4%, SRG = 16.3%, and PG = 10%	0.86 (LG), 0.86, (SRG), and 0.83 (PG)	–
Corral de Zurita and Leite, 2003	+18	EFA	Total = 50.8%	0.81(Total AGTQ), 0.82 (LG), 0.85 (SRG), and 0.76 (PG)	–
Valle et al., 2003b	+18	EFA	–	0.82 (Total AGTQ), 0.87 (LG), 0.87 (SRG), and 0.87 (PG)	–
Escurra et al., 2005	+18	–	–	0.94 (Total AGTQ), 0.85 (LG), 0.90 (SRG), and 0.86 (PG)	–
Inglés et al., 2009	12–16	CFA	Correlated three-factor model SRMR = 0.05; CFI = 0.90; GFI = 0.93; AGFI = 0.91; and RMSEA = 0.054	0.79 (LG), 0.74 (SRG), and 0.71 (PG).	–
Inglés et al., 2011	12–16	Factorial invariance	Correlated three-factors model adjusted by gender and age samples	–	–
Present study	12–16	–	–	0.86 (LG), 0.75 (SRG), and 0.89 (PG)	0.85 (LG), 0.83 (SRG), and 0.78 (PG)
**LASSI-HS**					
Weinstein and Palmer, 2002	9th grade	–	–	Attitude = 0.74, Motivation = 0.78, Time Management = 0.77, Anxiety = 0.82, Concentration = 0.82, Information Processing = 0.80, Selecting Main Ideas = 0.71, Study Aids Scale = 0.68 Self-testing = 0.74 and Test Strategies = 0.81	–
Olivarez and Tallent-Runnels, 1994	9th grade	EFA CFA	Total = 77% Correlated three factor model (GFI = 0.89; AGFI = 0.80; RMSR = 0.09)	Range = 0.70–0.82	–
Núñez et al., 1998	10–14	EFA	Eight factor model Total = 42.5%	Total LASSI-HS:.83 Range = 0.59–0.88	
Samuelstuen, 2003	15	CFA	Correlated three factor model (GFI = 0.94; AGFI = 0.89; RMSR = 0.08)	Range = 0.70–0.83	–
Stevens and Tallent-Runnels, 2004	7th–9th grade	CFA Factorial invariance	Correlated three factor model (CFI = 0.93; SRMR = 0.07) Factorial invariance was found across gender but not across ethnic groups	Range = 0.64–0.82 (total sample), 0.66–0.82 (boys sample), 0.71–0.82 (girls sample), 0.61–0.84 (Caucasian sample), and 0.70–0.83 (Hispanic sample).	–
García-Fernández et al., 2015	14–18	–	–	Range = 0.72–0.83	–
Delgado et al., 2018	12–16	–	–	Range = 0.68–0.82	–
Present study	12–16	–	–	Range = 0.71–0.82	Range = 0.75–0.86

*–, authors do not provide this information; EFA, Exploratory Factorial Analysis; CFA, Confirmatory Factorial Analysis; AGTQ, Achievement goals tendencies questionnaire; LG, Learning Goals; SRG, Social Reinforcement Goals; PG, Performance Goals; LASSI-HS, Learning and Studies Skills Inventory-High School Version.*

### Procedure

First, an interview was conducted with the school headmasters in order to inform about the purpose of the research and request their permission and collaboration. Subsequently, the written consent of the parents was required for their children to participate in the study. The questionnaires were administered collectively in the classroom and the investigators were present during the test administration in order to resolve any doubts and to emphasize the anonymous nature of the results obtained. An average of 10 min was used for the application of the AGTQ and 25 min for the LASSI-HS.

### Statistical Analyses

The profiles of academic goals were defined based on the different combinations of Learning Goals, Achievement Goals and Social Reinforcement Goals. A Latent Class Analysis (LCA), which has solved the problems of K-means clustering (Schreiber, 2017), was used in order to determine the number of profiles proposed. Subjects were first assigned to one class and then, they were to try to create the number of classes that researchers have considered. In order to choose the class that best represents the data, the lowest Bayesian Information Criteria (BIC) and an Entropy value closer to one were the fit indices used (Nyland et al., 2007; Schreiber, 2017; Smeets et al., 2017). ANOVAs were performed to analyze the significance of the differences between groups in the dimensions of learning strategies. In order to analyze the effect size of the eta-squared obtained (η^2^), the following Cohen’s rule was used (Miles and Shevlin, 2001): small (0.01 ≤ η^2^ ≤ 0.05), moderate (0.06 ≤ η^2^ ≤ 0.13) and large (η^2^ ≥ 0.14) Subsequently, in the analyses in which the differences were significant, *post hoc* tests were conducted to identify the groups in which the differences were established. The Scheffé method was chosen since it does not require that sample sizes be identical. Similarly, the effect size *d* (Cohen, 1988) was calculated to calculate the magnitude of the observed differences. Its interpretation is simple: small (0.20 ≤*d* ≤ 0.49), moderate (0.50 ≤*d* ≤ 0.79), and large (*d* ≥ 0.80) effect size. The data were analyzed using SPSS version 23.0.

## Results

### Academic Goal Profiles

**FIGURE 1:**
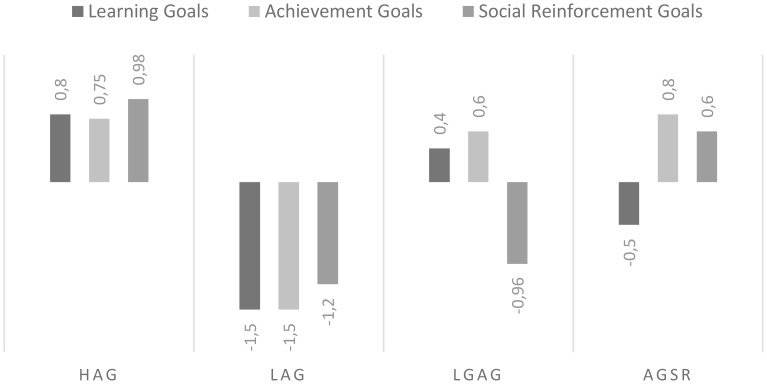
Graphic representation of the Latent Class Analysis solution. Cluster 1 (High Academic Goals; HAG), Cluster 2 (Low Academic Goals; LAG), Cluster 3 (Learning Goals and Achievement Goals; LGAG) and Cluster 4 (Achievement Goals and Social Reinforcement Goals; AGSR).

The LCA identified that the class consisting of four profiles with different levels of academic goals (see **Figure 1**) was the one that best suited the previously mentioned BIC and entropy criteria, as seen in **Table 3**. The first profile, HAG, included 668 students (32.28%) with high levels in all evaluated goals. The second profile, Low Academic Goals (LAG), consisted of 502 participants (24.26%) characterized by low levels in three goals academics. The third profile, with a predominance of Learning Goals and Achievement Goals (LGAG) classified 489 students (23.65%) and a fourth profile, with a predominance of Achievement Goals and Social Reinforcement (AGSR) consisted of 410 students (19.81%).

**Table 3:** Fit indices of the LCA values in bold revealing the best model fit.

Number of classes	BIC	Entropy
2 classes	15833.79	0.68
3 classes	14628.61	0.73
**4 classes**	**14372.113**	**0.77**
5 classes	15247.389	0.68
6 classes	15745.74	0.75

*BIC, Bayesian Information Criteria.*

### Inter-group Differences in Learning Strategies

Differences were found in learning strategies among the four academic goal profiles (p < 0.001) (see **Table 4**). The post-hoc contrasts show that students from the Learning Goals and Achievement Goals profile (LGAG) obtained significantly higher scores on the Attitude scale than students from the HAG, LAG and AGSR profiles, with effect sizes ranging from small to large (d = 0.32–0.80). Likewise, the students from the HAG profile obtained significantly higher scores on Attitude than the students from the LAG and AGSR profiles, with low effect sizes (d = 0.42–0.46, respectively). No statistically significant differences were obtained between the LAG and AGSR profiles.

**Table 4:** Means and standard deviations of the profiles and statistical significance.

	HAG profile		LAG profile		LGAG profile		AGSR profile		*F*_(3.2065)_	*p*	η^2^
	*M*	*SD*	*M*	*SD*	*M*	*SD*	*M*	*SD*			
Attitude	29.52	5.57	26.95	5.61	31.24	5.21	27.25	5.18	106.854	0.00	0.137***
Motivation	30.86	5.49	26.09	5.08	31.76	5.00	27.63	5.17	16.764	0.00	0.024*
Time Management	22.07	4.25	20.61	3.88	22.42	4.36	21.16	3.63	21.309	0.00	0.031*
Anxiety	27.53	5.49	24.59	5.37	26.73	5.30	27.11	4.97	39.375	0.00	0.055**
Concentration	27.44	6.10	24.95	5.71	28.65	6.13	25.31	5.32	81.453	0.00	0.108**
Information Processing	27.31	5.02	22.76	5.09	27.26	5.00	24.66	4.55	55.192	0.00	0.076**
Selecting Main Ideas	18.43	3.47	16.51	3.01	18.80	3.43	16.73	3.18	47.071	0.00	0.065**
Study Aids Scale	24.52	5.18	20.50	4.57	23.98	5.18	22.99	4.68	81.166	0.00	0.108**
Self-testing	27.02	4.93	22.39	4.67	26.75	4.87	24.58	4.41	37.888	0.00	0.053*
Test Strategies	28.51	5.78	27.48	4.82	30.13	5.33	26.63	5.08	61.122	0.00	0.083**

*HAG, High academic goals; LAG, Low academic goals; LGAG, Learning and achievement goals; AGSR, Achievement goals and social reinforcement goals; ^∗^small effect size; ^∗∗^moderate effect size; ^∗∗∗^large effect size.*

Group differences were also found for Motivation. Here, post hoc contrasts reveal that students from the LGAG profile obtained significantly higher scores on Motivation than students from the HAG, the LAG, and the AGSR profiles. The effect sizes ranged from small to large (d = 0.17–1.13). Likewise, students from the HAG profiles obtained significantly higher scores on Motivation than students from the LAG, and AGSR profiles with effect sizes ranging from moderate to large (d = 0.60–0.89, respectively). Differences were also found when contrasting the LAG and AGSR profiles, with the AGSR profile receiving significantly higher scores on Motivation than students from the LAG profile. However, the effect sizes were small (d = 0.30).

As for Time Management, here too, group differences were found. Students from the LGAG profile obtained significantly higher scores on Time Management than the LAG and AGSR profile. However, the effect sizes of these differences were small (d = 0.32–0.43, respectively). Also, students from the HAG profile obtained significantly higher scores in Time Management than students from the LAG and AGSR profiles with small effect sizes (d = 0.23–0.35, respectively). The remaining comparisons were not statistically significant.

Regarding anxiety, once again, group differences were found for the Anxiety scale. Students from the HAG, AGSR and LGAG profiles obtained significantly higher scores on Anxiety than students from the LAG profiles, with moderate effect sizes (d = 0.40; d = 0.49; d = 0.54, respectively). No statistically significant differences were found for the rest of the groups analyzed.

The post hoc contrasts show that students from the LGAG profile obtained significantly higher scores on Concentration than students from the HAG, LAG, and AGSR, with effect sizes ranging from low to moderate (d = 0.20–0.62). Moreover, the HAG profile received a significantly higher mean score than the LAG and AGSR profiles, however, the effect sizes were small (d = 0.31–0.20, respectively). No statistically significant differences were found between the LAG and AGSR.

Group differences were observed in the Information Processing scale. Here, the LGAG and HAG profiles obtained significantly higher scores on Information Processing than students from the LAG and AGSR profiles, with effect sizes ranging from moderate to large (d = 0.89–0.55). Likewise, students from the AGSR profile obtained significantly higher scores in Information Processing than students from the LAG profile, with effect sizes small (d = 0.40). No statistically significant differences were found between the LGAG and the HAG profiles.

Similarly, group differences were obtained for Selecting Main Ideas. The LGAG and HAG profiles obtained significantly higher scores on Selecting Main Ideas than students from the LAG and AGSR profiles, with moderate effect sizes (d = 0.51–0.70). No statistically significant differences were found between the earning Goals and Achievement Goals (LGAG) and HAG profiles or between the LAG and AGSR profiles.

As for the Study Aids scale, the post hoc contrasts show that students from the LGAG and HAG profiles obtained significantly higher scores than students from the LAG and AGSR profiles, with effect sizes ranging from small to large (d = 0.20–0.80). Also, students from the AGSR profiles obtained significantly higher scores on Study Aids than students from the LAG group, with moderate effect sizes (d = 0.54). No statistically significant differences were found between the LGAG and HAG profiles.

Similar results are obtained with respect to the Self-testing scale. The LGAG and the HAG profiles obtained significantly higher scores on the Self-testing scale than students from the LAG and AGSR profiles, with effect sizes ranging from small to large (d = 0.47–0.91). Also, students from the AGSR profile obtained significantly higher scores on Self-testing scale than students from the LAG profile, with small effect sizes (d = 0.49). No statistically significant differences were found between the LGAG and HAG profiles for the Self-testing scale.

Lastly, group differences were obtained on Test Strategies. The LGAG obtained significantly higher scores on Test Strategies than students from the HAG, LAG, and AGSR profiles, with effect sizes ranging from small to moderate (d = 0.29–0.67). Likewise, students from the HAG profile obtained significantly higher scores on Test Strategies than students from the AGSR profile, with small effect sizes (d = 0.39). No statistically significant differences were found between the HAG and LAG profiles or between the LAG and AGSR profiles.

## Discussion

### Interpretation and Discussion

The main goal of this work was to analyze the different combinations of goals and to define the academic goal profiles in a sample of Spanish high school students. Subsequently, the study examined whether significant differences exist between the obtained groups on the learning strategy dimensions. Thus for LCA, we identified four different motivational profiles: A profile with HAG, a second profile of LAG, a third profile with predominance of LGAG, and lastly, a profile with predominance of Achievement Goals and Social Reinforcement Goals (AGSR). The results coincide with previous investigations that have found groups of students with high scores on multiple goals, students with low scores on the different goals and profiles of students where one or several academic goals predominated (Linnenbrink, 2005; Daniels et al., 2008; Valle et al., 2010; Wormington et al., 2012; Hofer and Fries, 2016; Navas et al., 2016; Wormington and Linnenbrink-García, 2017; Clarence, 2018; Ning, 2018; Rameli et al., 2018). The results reveal that the majority of students are oriented toward multiple goals during their learning. That is, students use distinct motivations to take on different learning tasks.

Also, the results reveal statistically significant group differences in learning strategies. Academic goals set students’ behavioral intentions and, consequently, the regulation of students’ learning in a certain direction. Thus, generally speaking, it was found that students from the combined LGAG and HAG profiles used more learning strategies that those in the LAG and AGSR groups. These findings are consistent with studies that have reported that higher levels of motivation, cognitive strategies, self-regulation strategies and performance are seen in the group that combines high learning goals and high performance goals (Bouffard et al., 1995; Harackiewicz et al., 2000; Piñeiro et al., 2001; Valle et al., 2003b; Daniels et al., 2008; Ahmad and Bashir, 2009). Thus, Pintrich (2000) confirmed that learning-oriented students and those concerned about their performance have the same school adaptive pattern as those who are only learning-oriented. However, the author also states that this path is no longer equally adaptive in the case of those students only concerned about performance. Similarly, Daniels et al. (2008) obtained similar data in university students. The only students with maladaptive pattern were those called low motivation, while students with multiple goals (learning and performance oriented), mainly learning-oriented, and mainly performance-targeted show good and equivalent performance levels. Furthermore, it has also been found that the students who combine academic (learning and performance goals) and social goals use more learning strategies that the LAG and AGSR groups. Different research has shown that maintained social goals and successful peer relationships can help students to become more involved in the teaching and learning process, using more learning strategies and fulfilling better achievements (Wentzel, 2001; Mestre et al., 2006).

However, as shown in this study, LGAG use more specific learning strategies (attitude, motivation, concentration and test strategies) than students with HAG, with statistically significant differences between the two. Consequently, it can be suggested that not all benefits arise when students achieve multiple goals. Properly managing a wide variety of goals is more complex and may cause conflict and hinder the student’s task in certain cases (Kaplan and Maehr, 2007). Therefore, in order to achieve positive multiple goals, coordination is necessary so that attaining one goal does not block the achievement of another (Pintrich and Schunk, 2002).

On the other hand, it is noted that the LAG profile obtained the lowest average in the use of learning strategies. As mentioned in several investigations, this motivational pattern can adversely affect the implementation of learning strategies and academic performance (Ruthig et al., 2004; Valle et al., 2009).

### Conclusion and Added Values

This research has revealed that different academic and social goals are not independent, but have reciprocal effects. For example, in that they maintain social goals and successful peer relationships, they may encourage students to become more involved in the teaching and learning process and achieve better achievements (Wentzel, 2001; Mestre et al., 2006). Thus, when combined, good academic results may be achieved and these results are an indicator of capacity with their peers, aside from wanting to earn social approval which may lead to a higher levels of anxiety, a greater fear of failure and, consequently, poorer academic results (Valle et al., 2009). In fact, in this study, the HAG profile has higher anxiety levels than other groups, although these differences were only significant with respect to the LAG group. Thus, the search for mastery of a course as well as the demonstration of capacity, in comparison to their peers, may be associated with certain levels of anxiety (Suárez et al., 2005).

On the other hand, the results of this study confirm that students, in general, are oriented toward multiple goals during their learning process, being able to adapt successfully to different school situations. On the other hand, it is confirmed that students who use this motivational pattern, orienting themselves toward different goals in the function of the situation, optimize their teaching–learning process, using more learning strategies, which will affect their academic performance. This research highlights the need to study academic goals that are not considered mutually exclusive, obtaining a more objective and accurate scenario of school reality. Likewise, the need to not consider the learning goals as the most adequate for the student against the performance goals, being the combination of all of them, and the choice of one or the other depending on the school situation, the profile that provides a more effective scenario for student learning. However, although previous research has already corroborated these results, especially with university students, it is difficult to compare results, due to the use of different evaluation criteria and different types of academic goals.

### Limitations

This study has certain limitations that should be mentioned. First, the sample used (Spanish high school students) does not allow for the extrapolation of the results to other educational levels or to other countries. In addition, the evaluation of both constructs: academic goals and learning strategies, has been carried out using self-report instruments, so it may be necessary to complement this evaluation with other measurement instruments (interviews or perception of parents and teachers). On the other hand, the transverse nature of the design does not establish a causal relationship between the different variables considered in the study, so it may be of interest to carry out longitudinal studies to strengthen the results obtained.

Additionally, future studies should check the relationship between the different motivational profiles, the use of learning strategies and the student’s subsequent academic performance. The present research points out the relationship between motivational profiles and the use of certain learning strategies, and although it would be expected that students with a LGAG profile and HAG (those who use more learning strategies) will obtain a higher academic performance, this relationship has not been proven and it should be the subject of study in future research. In fact, Valle et al. (2009) found that the group with high generalized motivation obtained an inferior academic performance that the group with motivation toward learning and achievement.

### Theoretical Implications

Despite these limitations, this study provides a better view of the school reality with respect to the academic goals of high school students, corroborating how most students are oriented toward multiple academic goals during their teaching-learning process, which entails a greater use of learning strategies that will undoubtedly affect their adequate academic adjustment.

### Applications for Education Practice

Therefore, these results may be used for the development of strategies and intervention programs to promote the use of multiple academic goals in high school students. Thus, in order to improve the involvement and study strategies of students, school psychopedagogical intervention programs should detect students with maladaptive motivational profiles (LAG and AGSR) and include interventions aimed at developing their motivational orientations toward performance and learning goals. The results of this study also have an important practical implication for the teachers. They should promote the involvement of students toward learning and achievement together and reduce social comparison and competitiveness during the teaching process.

## Author Contributions

MM-M conceived of the study, participated in its design and coordination, and drafted the manuscript. BD participated in the drafting of the manuscript. RS performed a critical review of the manuscript and assisted with interpretation of the findings. CI assisted with the study conception and participated in the statistical analyses. JG-F participated in the design of the study, data interpretation, and assisted in drafting the manuscript. All authors read and approved the final manuscript.

## Conflict of Interest Statement

The authors declare that the research was conducted in the absence of any commercial or financial relationships that could be construed as a potential conflict of interest.
